# The Motor Optimality Score—Revised Improves Early Detection of Unilateral Cerebral Palsy in Infants with Perinatal Cerebral Stroke

**DOI:** 10.3390/children11080940

**Published:** 2024-08-04

**Authors:** Natascia Bertoncelli, Lucia Corso, Luca Bedetti, Elisa Muttini Della Casa, Maria Federica Roversi, Greta Toni, Marisa Pugliese, Isotta Guidotti, Francesca Miselli, Laura Lucaccioni, Cecilia Rossi, Alberto Berardi, Licia Lugli

**Affiliations:** 1 Neonatology Unit, Mother-Child Department, University Hospital of Modena, 41125 Modena, Italy; bertoncelli.natascia@aou.mo.it (N.B.); alberto.berardi@unimore.it (A.B.); 2 Department of Medical and Surgical Sciences for Mother, Children and Adults, Postgraduate School of Paediatrics, University of Modena and Reggio Emilia, 41121 Modena, Italy; 3 Bufalini Hospital, 47521 Cesena, Italy; 4 Psychology Unit, University Hospital of Modena, 41125 Modena, Italy; marisa.pugliese@unimore.it; 5 Pediatric Department, University Hospital of Modena, 41125 Modena, Italy; laura.lucaccioni@unimore.it

**Keywords:** arterial cerebral stroke, unilateral cerebral palsy, general movements assessment, segmental movement asymmetry

## Abstract

Background: Neonatal cerebral stroke includes a range of focal and multifocal ischemic and hemorrhagic brain lesions, occurring in about one of 3000 live births. More than 50% of children with neonatal stroke develop adverse outcomes, mainly unilateral cerebral palsy. Asymmetries in segmental movements at three months have been proven to be an early sign of CP in infants with unilateral brain damage. Recognition of additional early signs could enhance prognostic assessment and enable an early and targeted intervention. Aim: The aim of the study was to assess early signs of CP in infants with arterial cerebral stroke through the General Movements Assessment and the Motor Optimality Score—Revised (MOS-R). Method: Twenty-four infants born at term (12 females and 12 males) diagnosed with ACS, and 24 healthy infants (16 females and 8 males) were assessed. The GMs (fidgety movements) and MOS-R were assessed from videos recorded at 11–14 weeks of post-term age. Cognitive and motor outcomes were assessed at 24 months using the Griffiths III developmental quotient and Amiel-Tison neurological examination. The gross motor function classification system expanded and revised (GMFCS-E&R) was adopted to categorize CP. Results: Among infants with ACS, 21 (87.5%) developed unilateral CP. Most of them showed non-disabling CP (14 had GMFCS-E&R grade 1 [66.6%], 6 grade 2 [28.6%], and 1 grade 5 [4.8%]). Fidgety movements (FMs) were absent in 17 (70.8%), sporadic in 4 (16.7%) infants, and normal in 3 (12.5%). Segmental movement asymmetry was found in 22/24 (91.7%). According to the MOS-R, motor items (kicking, mouth movements), postural patterns (midline centered head, finger posture variability), and movement character (monotonous and stiff) were statistically different among infants with ACS and healthy infants. The MOS-R median global score was lower in the group with ACS compared to the control group (6 vs 26; *p* < 0.01). FMs, segmental movement asymmetry, and MOS-R global score were significantly correlated with abnormal outcome. MOS-R global scores less than or equal to 13 had 100% specificity and sensitivity in predicting GMFCS-E&R grade ≥ 2 CP in infants with ACS. Conclusions: The rate of CP was high among infants with ACS, but in most cases it showed low GMFCS-E&R grades. The study highlighted a significant correlation between MOS-R, together with absent FMs and unilateral CP in infants with ACS. Moreover, the MOS-R showed high sensitivity and specificity in the prediction of CP. Combined assessment of FMs and MOS-R could help to better identify infants at high risk of developing UCP in a population of infants with ACS. Early identification of precocious signs of unilateral CP is fundamental to providing an early individualized intervention.

## 1. Introduction

Perinatal stroke has been defined as a heterogeneous group of conditions in which there is a focal interruption of cerebral blood flow secondary to a thrombotic or embolic event in the arterial or venous system. The IPS generally occurs between 20 weeks of gestation and 28 days after birth, with an estimated incidence in term infants from 1:2300 to 1:5900 [1,2,3,4].

Arterial cerebral stroke (ACS) is the most common type of acute neonatal stroke, accounting for about 90% of cases. Most cases occur in term neonates, although ACS has also been attested in preterm infants [3]. The most commonly involved arterial branch is the middle cerebral artery (MCA) (90%), followed by the posterior cerebral artery (PCA) (9%) and the anterior cerebral artery (ACA) (1%), with the left cerebral hemisphere being the side most frequently affected. ACS is the leading cause of cerebral palsy (CP), particularly unilateral cerebral palsy (UCP). Predicting CP in newborns who have experienced an ACS involves various approaches and factors, including clinical assessments and neuroimaging [1,2,3,4]. One of the key methods used in predicting CP in newborns is the assessment of general movements (GMs) [5,6,7,8,9,10]. This method, developed by Prechtl HFR, involves the assessment of spontaneous movements of infants [5,6,7]. The quality of these movements, particularly during the early months of life, can be highly indicative of neurological integrity. Normal GMs involve the entire body in a variable sequence of movements that encompass arms, legs, neck, and trunk. They have a gradual beginning and end, they are fluent, elegant, and complex. GMs are classified based on the period during which they are observed, namely: writhing movements, present from term age (40 weeks of gestational age) until 4–6 weeks post-term (PT), and fidgety movements (FMs) which emerge gradually between 6 and 9 weeks PT and persist until 19–20 weeks PT. The absence of FMs or the presence of abnormal writhing movements has a high predictive value for CP. Specifically, the absence of FMs at 10–15-weeks PT is particularly concerning [5,6,7,8,9,10,11].

Nevertheless, the GM sensitivity-values for CP range from 95% to 98%. As a matter of fact, mild, usually unilateral CP has exceptionally been reported in infants who showed normal FMs [5,6,7,8,9,10,11,12,13,14]. To enhance the sensitivity and predictive power of this tool, additional assessments have been included such as the Motor Optimality Score—Revised (MOS-R) at 3–5 months. The MOS-R is a semi-quantitative tool used to assess in detail the quality of spontaneous movements and posture in infants, and it provides insight into later motor function. The MOS-R evaluates an infant’s motor repertoire in a detailed and structured manner at 3–5 months, helping to identify those at high risk of CP and other neurodevelopmental disorders. The MOS-R is typically scored on a scale that reflects the degree of normality and optimality of the infant’s movement and postural patterns. According to the literature, infants with an MOS-R ≤ 14 with absent FMs should be confidently referred to targeted early treatment programs during the period of greatest neuroplastic changes.

Thus, the MOS-R score provides additional information for the evaluation of FMs regarding age-specific motor and postural patterns. Segmental movements (SMs) are one of the movement patterns in the MOS-R and highlight asymmetries in infants with absent FMs. SMs are moderate-speed movements at the wrist involving rotation, palm flexion–extension, and radial and ulnar flexion. Several studies have suggested that an asymmetry in SMs is an early predictive sign of unilateral CP. An asymmetry can be clinically significant, even if brain imaging is negative for lesions [12,13,14,15].

The MOS-R may be particularly relevant in the context of infants with ACS to predict the risk of CP [16,17,18,19]. Early identification of at-risk infants through the MOS-R may allow for timely and targeted interventions, which can improve long-term motor and developmental outcomes. Anyway, at present, there are no studies specifically investigating the role of the MOS-R in predicting CP in infants with ACS. Indeed, our study aims to assess GMs and MOS-R in infants with ACS to improve the detection of early signs of CP.

## 2. Aims of the Study

The aims of the study are:

(1) To compare GMs and the MOS-R in infants with ACS and in healthy full-term infants;

(2) To identify early signs of unilateral CP in infants with ACS using GMs, SMs assessment, and the MOS-R.

## 3. Materials and Methods

### 3.1. Study Design

The study was a longitudinal, retrospective, single-center study performed in the Neonatal Intensive Care Unit (NICU) of the University Hospital of Modena, Italy.

### 3.2. Subjects

Full-term infants diagnosed with ACS through brain MRI were recruited from 1 January 2000 to 31 December 2021 and followed up to 24 months. Infants with venous or mixed cerebrovascular lesions, and those with incomplete follow-up, were excluded from the study. Healthy full-term infants with normal motor and cognitive outcomes at 24 months were also recruited in the same study period as a control group. The study was approved by the Ethics Committee Area Vasta Emilia Nord (AOU 0004487/21, 10/02/2021). This research received no funding.

### 3.3. General Movements Assessment and MOS-R

At the age of 10–13 weeks PT, both case and control groups were videorecorded in supine position, dressed in a bodysuit, for 2 min during quiet wakefulness. The camera was positioned above the infant at an angle of about 45 degrees. Three evaluators (NB, GT, and LLuc), certified in global and detailed assessment of GMs, performed the analysis by reviewing enrolled patients’ videos. In the case of disagreement between the three evaluators, an agreement was reached after discussion. The assessment included a global analysis based on the FMs assessment, according to Prechtl’s method, and the detailed analysis based on the MOS-R evaluation [15,16,17,18,19,20]. The MOS-R included five score subcategories: i. quality of fidgety movements, ii. quality of movement patterns, iii. age-adequate movement repertoire, iv. quality of postural patterns, v. movement character (Supplementary Table S1). Total MOS-R scores ranged from 5 to 28 (Supplementary Table S2). Each motor and postural pattern was assessed as normal or atypical based on the definition provided in the MOS-R manual (Supplementary Table S3). Additionally, the age-adequate movement repertoire score was also defined. The Motor Optimality List (including FMs, observed movement patterns, age-adequate movement repertoire, observed postural pattern, and movement character) was assessed, and the MOS-R score was calculated for each infant. Moreover, SMs were counted in infants with absent or sporadic FMs. The frequency per minute and the Asymmetry Index (AI) were calculated [13] according to the following equation: (*vCM* − *vIM*)/(*vCM* + *vIM*). The vCM is the frequency per minute of SMs in the contralateral hand, and vIM is the frequency per minute of movements in the ipsilateral hand. The resulting AI ranged from −1 to +1, and a negative score indicates fewer SMs in the contralateral hand.

### 3.4. Brain Magnetic Resonance Imaging (MRI)

Brain MRI was performed on a 1.5 Tesla system (Philips Intera 1.5-T Medical Systems, Best, The Netherlands). Diffusion-, T_1_-, and T_2_-weighted images were obtained in the axial, coronal, and sagittal planes in sections of 5 mm. In all cases, the neonatal MRI was performed within the first week of life. ACS were classified according to the distribution of the lesion into (a) involvement of the main branch of a cerebral artery (anterior, middle, posterior cerebral artery), (b) involvement of a cortical branch, (c) involvement of the boundary zones between cerebral arteries [13].

### 3.5. Outcome Assessment

Neurodevelopmental follow-up was performed by a multidisciplinary team comprising an experienced neonatologist in neuro-developmental neurology, a child psychologist, and a physiotherapist, as previously detailed [21,22,23]. To ensure adherence, parents received timely telephone reminders for appointments. Infants underwent evaluation using the Amiel-Tison neurological assessment [24,25] and the Griffiths Mental Developmental Scales (GMDS-R) [26]. The GMDS-R (0–2 years) provided a general development quotient (DQ) for infants’ abilities and five subscale quotients (locomotor, eye and hand co-ordination, personal and social, hearing and language, and cognitive performance). The Amiel-Tison examinations included a series of assessments at different ages (0–6 years), allowing the clinician to track signs of permanent brain damage. Based on the neurological examination at 24 months, the outcome was categorized as either normal or abnormal due to CP [24,25]. Cerebral palsy was defined as a permanent disorder of the development of movement and posture, causing activity limitations [27]. Infants with CP were classified according to the Gross Motor Function Classification System—Expanded and Revised (GMFCS-E&R) [28,29].

Medians and ranges were calculated for continuous variables, while categorical variables were expressed as counts and percentages. The χ^2^ test was used for categorical variables, while continuous variables were compared using the Mann–Whitney test. Risk factors for CP were evaluated with univariate regression analysis. The ROC curve analyses were also evaluated. A *p* value < 0.05 was considered statistically significant. The statistical analysis was performed using MedCalc statistical software (version 22.032).

## 4. Results

**Figure 1:**
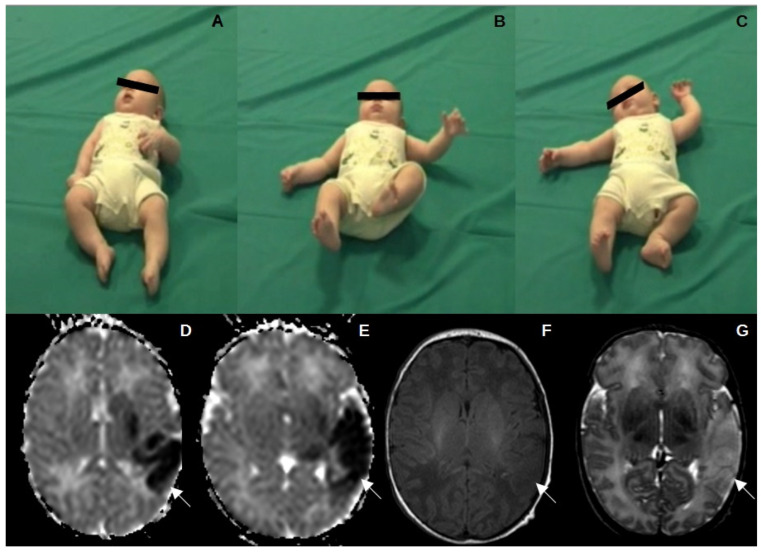
Absent FMs, SMs, and postural asymmetry (**A**–**C**) in a patient (case 15) with left MCA stroke, who developed right UCP. Cerebral MRI performed at 5 days of life showed diffusion-weighted (**D**,**E**), T1 (**F**), and T2 (**G**) abnormalities in the left MCA cortical and subcortical areas.

Twenty-four infants with ACS (12 female, 12 male, median gestational age 40 weeks, median birth weight 3127 g) and 24 control cases (16 female, 8 male, median gestational age 40 weeks, median birth weight 3430 g) were evaluated. All but one infant with ACS (95.8%) presented with neonatal seizures, while one infant presented with clinical features of neonatal encephalopathy. Based on brain MRI, the ACS-affected arterial branch was the MCA in 15/24 cases (62.5%), the posterior cerebral artery (PCA) was in 6/24 (25%), both MCA and PCA were in one infant (4.2%), and both ACA and MCA were in two infants (8.3%). Among 15 cases with MCA involvement, four infants (26.7%) had involved superficial and deep branches of the MCA, seven (46.6%) the anterior branch of the MCA, and four (26.7%) the posterior branch of the MCA. The right hemisphere was affected in 12 cases (50%), and the left hemisphere in 12 infants (50%). Twenty-one (87.5%) infants developed CP, while three (12.5%) had normal outcomes at 24 months (Table 1, Figure 1). Among the 21 infants with CP, 3 (14.3%) had a DQ < 70 and 1 (4.8%) had a DQ between 70 and 85, while the 17 remaining infants (80.9%) had a DQ within normal range (Table 1). All 24 control infants had normal neurodevelopmental outcomes.

### 4.1. GMs Assesment

Seventeen (70.8%) infants with ACS had absent FMs, four (16.7%) had sporadic FMs, and three (12.5%) had normal FMs (Table 1). In the control group, all infants had normal FMs.

### 4.2. Movement Patterns

Table 2 shows movement patterns according to the MOS-R in the two groups. A significant difference was observed for kicking (*p* < 0.0147), mouth movements (*p* < 0.0001), and atypical tongue movements (*p* = 0.0094).

Atypical: when repetitively occurring or asymmetric or stiff according to the observed movement pattern (Table S3).

### 4.3. Postural Patterns

Table 3 shows postural patterns according to the MOS-R. A significant difference was found for head-centered (*p* = 0.0002) and for variability of finger postures (*p* = 0.0001).

Atypical: each postural pattern has a specific atypicality. Table S3 describes each atypical postural pattern.

### 4.4. Movement Character

Table 4 shows movement character according to the MOS-R. Smooth and fluent character of movement was absent in infants with ACS, whereas 22/24 (91.7%) of healthy infants exhibited smooth and fluent movements (*p* < 0.0001). A monotonous, jerky, and stiff movement character was more frequent in infants with ACS compared to healthy infants (*p* < 0.0001, *p* = 0.0012, and *p* = 0.0141, respectively).

### 4.5. Motor Optimality List

Table 5 shows the motor optimality list in the case and control groups. In the case group, 3/24 (12.5%) had an age-adequate movement repertoire, 3/24 (2.5%) a reduced movement repertoire, and 18/24 (75%) an absent movement repertoire for their age. In the control group, 14/24 (58.3%) had a reduced movement repertoire, 7/24 (29.2%) an age-adequate movement repertoire, and 3/24 (12.5%) an absent age-adequate movement repertoire (*p* < 0.01). In the case group, 17/24 (70.8%) exhibited a predominance of atypical movement patterns, 2/24 (8.3%) an equal number of normal and atypical movements, and 5/24 (20.9%) a predominance of normal movement patterns. In the control group, all infants exhibited a predominance of normal movement patterns (*p* < 0.01). Among 24 infants with ACS, 18 (75%) exhibited a predominance of atypical postural patterns, 2 (8.3%) an equal number of normal and atypical postural patterns, and 4 (6.7%) a predominance of normal postural patterns. In the control group, 17/24 (70.8%) presented a predominance of normal postural patterns, 4/24 (16.7%) an equal number of normal and atypical postural patterns, and 3/24 (2.5%) a predominance of atypical postural patterns (*p* < 0.01). In the case group, the median MOS-R score was 6 (CI 6–9), while in the control group it was 26 (CI 25–26) (*p* < 0.0001) (Table 5). Among 24 cases with ACS, 22 (91.6%) had an MOS-R score < 20, indicating the need for early intervention (Table 1).

### 4.6. Neurodevelopmental Outcome and Prognostic Factors

**Figure 2:**
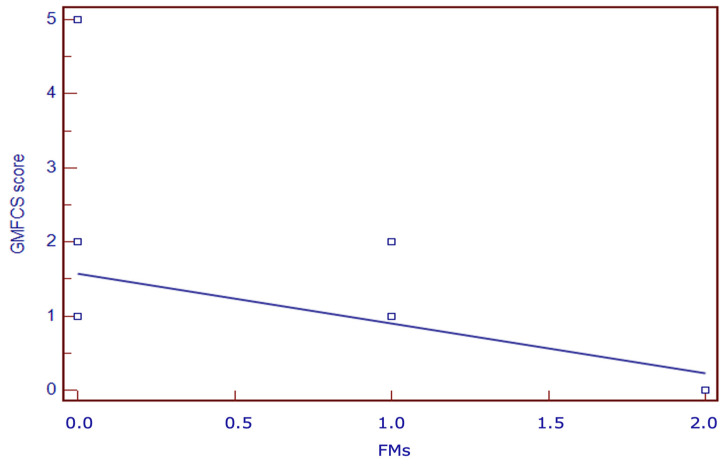
Linear regression between FMs and GMFCS-E&R.

**Figure 3:**
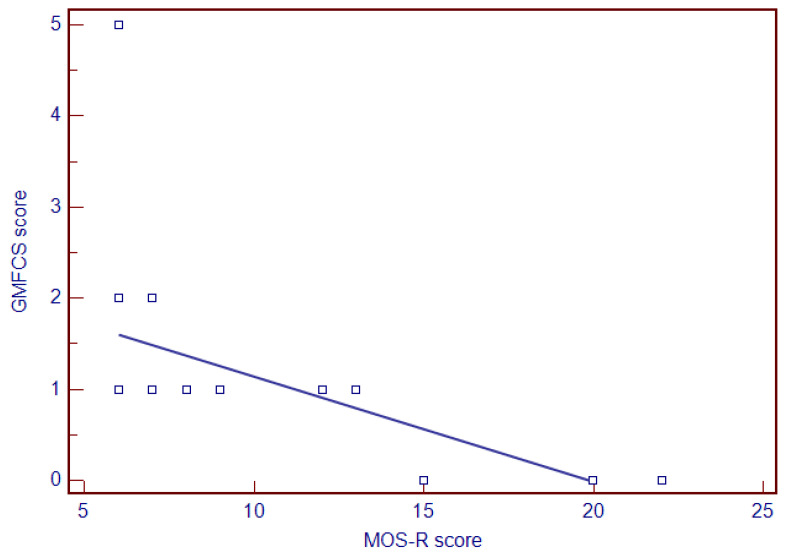
Linear regression between MOS-R and GMFCS-E&R.

**Figure 4:**
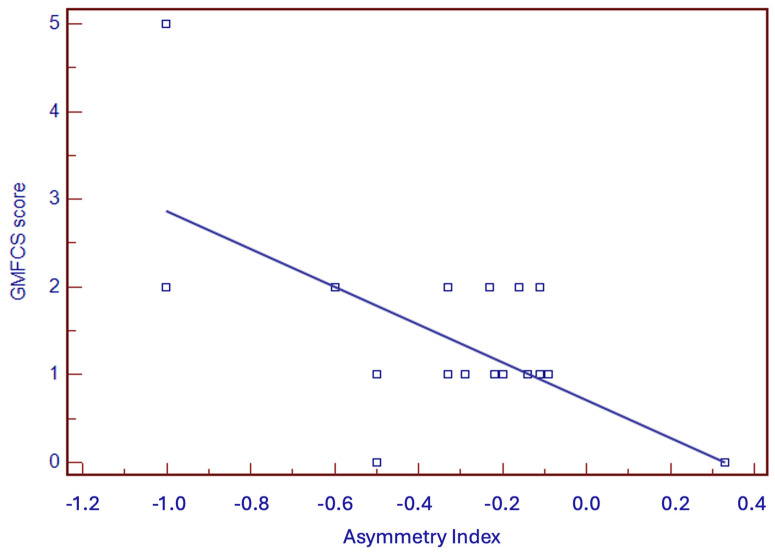
Linear regression between Asymmetry Index and GMFCS-E&R.

Among the 24 infants with ACS, 21 developed unilateral CP (87.5%), of whom 6 had GMFCS-E&R > 1 (33.3%) (Table 1). Among 22 infants with an MOS-R score < 20, 21 (95.5%) developed UCP, while one case (MOS-R = 15) had a normal outcome. All 22 infants with absent FMs presented a negative AI, and 21 of them (95.5%) developed UCP. In a univariate regression analysis, the MOS-R, AI, and FMs correlated significantly with the degree of PC (*p* < 0.01) (Figure 2, Figure 3 and Figure 4), while the occluded arterial branch did not. Sensitivity, specificity, and positive and negative predictive values of different variables (MOS-R, AI, FMs, arterial branch) in the early detection of GMFCS-E&R grade ≥ 2 CP are presented in Table 6. FMs and MOS-R < 13 resulted the best predictor of CP in infants with ACS.

## 5. Discussion

Targeted interventions are widely used to drive neuroplasticity and aid recovery following neurologic insult, including from CP. As evidence supports maximal neuroplasticity early in life with mechanisms unique to the developing brain, early identification of infants who may benefit from targeted interventions is thought to optimize functional recovery. Several assessment methods have proven effective in the early identification of infants who will develop CP, including the Hammersmith Infant Neurological Examination (HINE) and GMs. The HINE, a widely available and implemented standardized exam for children aged 2–24 months, provides optimality scores at different ages. The HINE total score has good predictive value for CP in high-risk infants at 3 months, with a cut-off score of 57. A total HINE asymmetry score (HINE AS) is obtained by summing the number of items with clinical differences between right and left sides. Among children with HINE total scores in the normal range, total HINE AS > 5 at 9 months can distinguish children with UCP from those with normal development. The Hand Assessment for Infants (HAI) is an additional instrument providing information about the quality or degree of impairment of upper extremity function in infants 3–12 months of age [30,31,32].

The fundamental significance of abnormal GMs as an early sign of brain dysfunction and CP is well known. Several studies [12,13,14] have shown the possibility of making an early prediction of neurological outcomes in different populations of infants at risk for UCP, such as preterm or term infants with ACS, through the observation of spontaneous motility. Guzzetta et al. [13] demonstrated that infants with ACS, who subsequently developed UCP, showed a reduction in distal SMs on the side contralateral to the lesion, in addition to abnormal GMs, when assessed around 3 months of age. This was not seen in infants with lesions, who showed a normal neurological outcome [13]. SMs are one of the movement patterns in the MOS-R, a semi-quantitative tool used to assess in detail the quality of spontaneous movements in infants, particularly when predicting the risk of CP. As the detection of additional early signs could enhance the prognostic assessment and enable an early and targeted intervention, the MOS-R may be particularly relevant in the context of infants with ACS to predict the risk of CP. To the best of our knowledge, there have been no studies that investigated specifically the role of the MOS-R in predicting CP in infants with ACS. A single pilot study investigated the role of the General Movement Optimality Score (GMOS) to detect early signs of motor disorder in infants with ACS [33]. The GMOS is a metric designed to evaluate in detail the quality of movements during the writhing period. That pilot study included 27 infants with a diagnosis of ACS, 8 of whom developed CP. As might be expected, the CP group had a lower GMOS than the non-CP group. In particular, the authors found significant contralesional differences in the distal rotatory components of the upper limbs, and tremulous movement of the lower limbs, between the two groups. No significant difference was found in the ipsilesional limbs between the two groups, but the score of global and contralateral limb showed significant differences [33]. Be that as it may, the GMOS includes many items, making it quite difficult to adopt in clinical practice. The MOS-R is designed to provide a practical and efficient way to assess in detail movement and postural patterns in clinical settings. The MOS-R focuses on key metrics that are crucial for evaluating movement while being easier to measure and interpret. Moreover, the MOS-R is used at a particularly crucial age for prognostic evaluation—the FMs period. In clinical practice, the adoption of the MOS-R may allow the early identification of infants at risk of CP in order for them to receive timely and targeted interventions, which can improve long-term motor and developmental outcomes. Indeed, our study evaluated the role of GMs and MOS-R in predicting CP in infants with ACS, showing that the adoption of MOS-R in addition to GMs improves early detection of neurological impairment. Our case-control comparison highlighted the potential of the MOS-R score to provide additional information regarding age-specific motor and postural patterns. According to the MOS-R, there were statistical differences in the motor patterns (kicking, mouth movements), postural patterns (head centered in midline, variability of finger posture), and movement character (monotonous and stiff) between infants with ACS and healthy infants. In the case group, the character of movement was predominantly monotonous, jerky, and stiff, with no infants exhibiting smooth and fluent movements, which were mostly present in the control group. Furthermore, 75% of infants with ACS had an absent age-adequate movement repertoire, compared to 12.5% in the control group. Moreover, the MOS-R median global score was significantly lower in the group with ACS compared to the control group. Among infants with ACS, 91.6% had an MOS-R score < 20, indicating the need for early intervention. In fact, 95.5% of infants with an MOS-R score < 20 developed unilateral CP. In the group of infants with ACS, the rate of unilateral CP was high (87.5%), but most infants (66.7%) presented mild and non-disabling CP. In the univariate regression analysis, the MOS-R score, the AI, and the FMs correlated significantly with the degree of CP. All infants who developed unilateral CP had absent or sporadic FMs, and had an MOS-R ≤ 13. The MOS-R and FMs presented maximum sensitivity, specificity, and positive and negative predictive values in the early detection of unilateral CP with a GMFCS-E&R grade ≥ 2.

This study had several limitations. The main limitation was its small sample size, but ACS is a rare condition and sometimes diagnosed after the first month of life, when motor impairment signs are already evident. Multicentric studies recruiting a larger number of infants with a confirmed diagnosis of ACS, and a similar follow-up, would be desirable. A second limitation is that, due to the small number of patients, cerebral MRI was classified based on the major arterial branch, but the involvement of subcortical structures such as the internal capsule, basal ganglia, thalamus, and brainstem was not considered. Finally, the HINE was not used in this study, and a comparison between MOS-R and HINE is lacking, but this could be the subject of future studies.

Conversely, our research has several strengths: it consisted of a homogeneous case series, all with MRI-documented ACS analyzed with different functional assessment tools (GMs, SMs, MOS-R) and compared with a control group. Thus, this study highlighted a significant correlation between MOS-R and UCP in infants with ACS.

## 6. Conclusions

The present study confirmed a correlation between absent FMs, together with asymmetries in SMs, and UCP in infants with ACS. The case-control comparison underscored the potential of the MOS-R to provide additional insights into age-specific motor and postural patterns. Moreover, the MOS-R showed high sensitivity and specificity in the prediction of UCP. Therefore, a combined assessment of FMs and MOS-R could help to better identify infants at high risk of developing UCP in a population of infants with ACS. Early identification of precocious signs of UCP is fundamental to providing an early individualized intervention.

## Figures and Tables

**Table 1:** Type of lesion, outcome, fidgety movements, MOS-R score, Griffiths DQ, and GMFCS score.

Infants	Type of Lesion	Outcome	Fidgety Movements	MOS-R Score	Asymmetry Index	Griffiths DQ	GMFCS Score
1	Right PCA	Left UCP	Absent	6	−1	69	2
2	Right PCA	Left UCP	Sporadic	13	−0.29	94	1
3	Left MCA (post)	Right UCP	Absent	9	−0.33	97	1
4	Left MCA (ant)	Right UCP	Absent	6	−0.33	115	2
5	Right MCA (ant)	Left UCP	Absent	6	−0.23	103	2
6	Left MCA + ACA	Right UCP	Absent	6	−0.11	49	2
7	Right MCA_(ant)	Normal	Normal	15	−0.5	106	0
8	Left MCA (ant)	Right UCP	Absent	7	−0.2	115	1
9	Left MCA	Right UCP	Absent	6	−1	48	5
10	Right MCA (ant)	Left UCP	Absent	6	−0.33	103	1
11	Left MCA (post)	Right UCP	Absent	6	−0.22	105	1
12	Right MCA (ant)	Left UCP	Absent	8	−0.2	94	1
13	Left MCA (ant)	Right UCP	Sporadic	6	−0.6	105	2
14	Right MCA (post)	Left UCP	Absent	6	−0.5	89	1
15	Left MCA	Right UCP	Absent	7	−0.16	110	2
16	Left MCA	Normal	Normal	22	0.33	104	0
17	Right MCA/PCA	Left UCP	Absent	12	−0.09	105	1
18	Left MCA (post)	Right UCP	Absent	6	−0.2	112	1
19	Left MCA	Right UCP	Absent	12	−0.29	80	1
20	Right PCA	Normal	Normal	20	0.33	112	0
21	Right PCA	Left UCP	Absent	6	−0.14	100	1
22	Right MCA + ACA	Left UCP	Sporadic	6	−0.14	93	1
23	Left PCA	Right UCP	Absent	6	−0.11	88	1
24	Right PCA	Left UCP	Sporadic	6	−0.14	105	1

PCA: posterior cerebral artery; MCA: middle cerebral artery; ACA: anterior cerebral artery; ant: anterior branch; post: posterior branch; UCP: unilateral cerebral palsy.

**Table 2:** Movement patterns according to the MOS-R.

Movement Patterns	Cases		Controls		Total	*p*-Value
	N (%)	A (%)	N (%)	A (%)	22	
Swipes	8/9 (89)	1/9 (11)	13/13 (100)	0	26	0.8499
Wiggling–Oscillating	5/7 (71.4)	2/7 (28.6)	19/19 (100)	0	22	0.1106
Kicking	2.6 (33.3)	4/6 (66.7)	15/16 (93.8)	1/16 (6.2)	8	0.0147
Excitement Bursts	0	1/1 (100)	77 (100)	0	20	0.2254
Smiles	5/8 (62.5)	3/8 (37.5)	12/12 (100)	0	39	0.0966
Mouth Movements	4/17 (23.5)	13/13 (76.5)	22/22 (100)	0	12	<0.0001
Tongue Movements	0	11/11 (100)		1/1 (100)	5	0.0094
Side-to-side Movement of the Head	0	5/5 (100)	0	0	NA	NA
Hand-to-Mouth Contact	3/3 (100)	0	1/1 (100)	0	4	0.6171
Hand-to hand Contact	3/3 (100)	0	5/5 (100)	0	8	0.7237
Fiddling	5/7 (71.4)	2/7 (28.6)	16/16 (100)	0	23	0.1517
Reaching	0	0	0	0	0	NA
Foot-to-Foot Contact	7/14 (50)	7/14 (50)	5/6 (83.3)	1/6 (16.7)	20	0.37
Legs lift	0	0	1/1 (100)	0	1	NA
Hand-to-toe Contact	0	0	1/1 (100)		1	NA
Segmental Movement of Wrists		22/22 (100)		0	22	NA
Arching	2/3 (66.7)	1/3 (33.3)	3/3 (100)	0	6	1
Rolling to side	0	2/2 (100)	2/2 (100)	0	4	0.3173
Visual Exploration	7/7 (100)	0	17/17 (100)	0	24	0.0662
Hand Regard	0		5/5 (100)		5	NA
Head Anteflexion	0	0	0	0	0	NA
Circular Arm Movements		0		0	0	NA
Almost no Leg Movements		0		0	0	NA

**Table 3:** Postural patterns according to the MOS-R.

Postural Patterns	Cases		Controls		Total	*p*-Value
	N (%)	A (%)	N (%)	A (%)		
Head-Centered	6/24 (25)	18/24 (75)	20/24 (83.3)	4/24 (16.7)	48	0.0002
Body Symmetry	4/24 (16.7)	18/24 (83.3)	3/24 (12.5)	21/24 (87.5)	48	1
Asymmetric Tonic Neck (ATN) Posture	19/24 (79.2)	5/24 (20.8)	24/24 (100)		48	0.0588
Flat Posture		1 (100)		0	1	NA
Variability of Finger Posture	7/24 (29.2)	17/24 (70.8)	21/23 (91.3)	2/23 (8.7)	47	0.0001
Predominant Fisting		6/6 (100)		2/2 (100)	8	0.2888
Synchronized Opening and Closing of Fingers		0		0	0	NA
Finger Spreading		6/6 (100)		2/2 (100)	8	0.2888
Asymmetry of Finger Posture		10/10 (100)		3/3 (100)	13	0.961
Hyperextension of Neck		0		0	0	NA
Hyperextension of Trunk		0		0	0	NA
Extended Arms		2/2 (100)		0	2	NA
Extended Legs		0		0	0	NA

**Table 4:** Movement character according to the MOS-R.

Movement Character	Cases		Controls		Total	*p*-Value
	Yes (%)	No (%)	Yes (%)	No (%)		
Smooth and Fluent	0	24/24 (100)	22/24 (91.7)	2/24 (8.3)	48	<0.0001
Monotonous	22/24 (91.7)	2/24 (8.3)	1/24 (4.2)	23/24 (95.8)	48	<0.0001
Jerky	12/24 (50)	12/24 (50)	1/24 (4.2)	23/24 (95.8)	48	0.0012
Stiff	7/24 (29.2)	17/24 (70.8)	0	24/24	48	0.0141
Predominantly Slow	2/24 (8.3)	22/24 (91.7)	0	24/24 (100) (100)	48	0.4701
Predominantly Fast	2/24 (8.3)	22/24 (91.7)	0	24/24 (100)	48	0.4701

**Table 5:** Motor Optimality List in the two groups.

Motor Optimality List	Cases	Controls	*p*-Value
	Median (IC)	Median (IC)	
Fidgety Movements	1 (1–1)	12 (12–12)	<0.0001
Observed Movement Patterns	1 (1–2)	4 (4–4)	<0.0001
Age-Adequate Movement Repertoire	1 (1–1)	2 (2–4)	0.0005
Observed Postural Pattern	1 (1–1)	4 (2–4)	0.0001
Movement Character	2 (2–2)	4 (4–4)	<0.0001
MOS-R Score	6 (6–9)	26 (25–26)	<0.0001

**Table 6:** Sensitivity, specificity, and positive and negative predictive values of different variables in prediction of cerebral palsy.

	Sensitivity (%) (CI 95%)	Specificity (%) (CI 95%)	PPV (%)	NPV (%)	ROC (CI 95%)
**MOS-R (<13)**	100 (83.7–100)	100 (30.5–100)	100	100	1 (0.86–1)
**AI (<−0.09)**	100 (83.7–100)	66.7 (11.6–94.5)	95.5	100	0.72 (0.50–0.88)
**Arterial branch (MCA)**	71.4 (41.9–91.4)	33.3 (55.5–88.4)	83.3	20	0.52 (0.27–0.76)
**Fidgety Movements**	100 (83.7–100)	100 (30.5–100)	100	100	1 (0.86–1)

PPV (positive predictive value); NPV (negative predictive value); CI (confidence interval).

## Data Availability

The data presented in this study are available on request from the corresponding author. The data are not publicly available due to privacy.

## Supplementary Materials

The following supporting information can be downloaded at: https://www.mdpi.com/article/10.3390/children11080940/s1, Table S1: Assessment of the Motor Optimality Score Revised; Table S2: MOS-R categories; Table S3: MOS-R: definition and criteria for scoring; Table S4: Age-adequate movement repertoire score.
