# Supplementary material for: The Motor Optimality Score—Revised Improves Early Detection of Unilateral Cerebral Palsy in Infants with Perinatal Cerebral Stroke
Source: Children (Basel). 2024 Aug 4;11(8):940. doi: 10.3390/children11080940 (PMC11352565; doi:10.3390/children11080940)
Supplement: Supplementary file 1 [file children-11-00940-s001.zip › children-3142311-supplementary.pdf]

Supplementary Table S1: Assessment of the Motor Optimality Score Revised

The Motor Optimality Score for 3- to 5-Month-Old Infants – Revised

Christa Einspieler and Arie Bos for the GM Trust 2000, 2019

Einspieler et al., submitted to J Clin Med 2019

Name: ....

Date of Birth: Gestational Age at Birth: Birth Weight:

Recording Date: Postmenstrual / Postterm Age:

Fidgety Movements (N, normal; A, atypical):

N Fidgety Movements
A Abnormal Exaggerated
A Absent
N A Sporadic (age-specific)

Observed Movement Patterns (N, normal; A, atypical): normal atypical

N A Swipes
N A Wiggling-Oscillating
N A Kicking
N A Excitement Bursts
N A Smiles
N A Mouth Movements
A Tongue Movements
N A Side-to-Side Movements of the Head
N A Hand-to-Mouth Contact
N A Hand-to-Hand Contact
N A Fiddling
N Reaching
N A Foot-to-Foot Contact
N A Legs Lift
N Hand-to-Toe Contact
A Segmental Movements of Fingers and Wrists
N A Arching
N A Rolling to Side
N A Visual Exploration
N Hand Regard
N A Head Anteflexion
A Circular Arm Movements
A Almost No Leg Movements
A ______ add

Observed Postural Patterns (N, normal; A, atypical): normal atypical

N A Head Centered
N A Body Symmetry
N A Asymmetric Tonic Neck (ATN) Posture
A Flat Posture
N A Variability of Finger Postures
A Predominant Fisting
A Synchronized Opening and Closing of Fingers
A Finger Spreading
A Asymmetry of Finger Post.
A Hyperextension of Neck
A Hyperextension of Trunk
A Extended Arms
A Extended Legs
A ______ add

Movement Character:

N Smooth and Fluent
A Monotonous
A Jerky
A Stiff
A Tremulous
A Cramped-Synchronized
A Predominantly Slow
A Predominantly Fast
A ______ add

Motor Optimality List:

i.	Fidgety Movements + ++, * **	normal	❑	12
		abnormal exaggerated	❑	4
	±	absent / sporadic	❑	1
ii.	Observed Movement Patterns	N > A	❑	4
		N = A	❑	2
		N < A	❑	1
iii.	Age-Adequate Movement Repertoire (do not consider fidgety movements)	present	❑	4
		reduced	❑	2
		absent	❑	1
iv.	Observed Postural Patterns	N > A	❑	4
		N = A	❑	2
		N < A	❑	1
v.	Movement Character	smooth and fluent	❑	4
		abnormal but not CS	❑	2
		cramped-synchronized (CS)	❑	1

Motor Optimality Score (MOS): from 28 to 5

Supplementary Table S2 MOS-R categories (Crowle et al 2023)

MOS-R score	Optimality category	Intervention indication category
25-28	Optimal	Referral to early intervention may not be indicated, if no concerns on clinical presentation and other assessment. *If upper limb asymmetry is identified, further assessment, monitoring and intervention should be carried out
20-24	Mildly reduced optimality	
9-19	Moderately reduced optimality	Referral to early intervention is recommended (if not already engaged)
5-8	Severely reduced optimality	

Supplementary Table S3. MOS-R: definition and criteria for scoring

Movement Pattern	Definition
**Swipes**	Ballistic-like movements with a sudden onset but fluid course and smoothoffset; can go in downward or upward direction; most noticeable in extended arms; but also in partially or fully extended legs; large amplitudeand high speed.<br><br>Score atypical if repetitively occurring in more than one third of the observation time.
**Wiggling-Oscillating Movements**	Oscillatory, uniplanar movements; most noticeable in partially or fully extended arms but from time to time also in partially extended legs, with afrequency of 2–3 Hz; small amplitude and moderate speed.<br><br>Score atypical if repetitively occurring in more than one third of the observation time.
**Kicking**	Can occur in a single leg and/or as simultaneous bilateral kicking with a fast flexion phase followed by a slower extension phase with decouplingof hip, knee and ankle.<br>Score atypical if monotonous and/or coupling of hip, knee, and ankle is observed such that all joints tend to flex or extend in temporal synchrony.
**Excitement Bursts**	Wiggling-oscillating movements and/or swipes co-occur with kicking andare accompanied by a pleasurable and excited facial expression.<br>Score atypical if monotonous and without pleasure mimic.
**Smiles**	Score atypical if awkward and frozen.
**Mouth Movements**	Are variable and usually related to vocalization (cooing) and/or non-nutritive sucking.<br>Score atypical if opening and closing occur repetitively.
**Tongue Movements**	Score atypical if tongue protrusion is repetitive and/or long-lasting.
**Side-to-Side Movements of the Head**	The head crosses the midline smoothly and fluently. Do not mark if thehead moves only from midline to side and back.<br>Score atypical if repetitive.
**Hand-to-Mouth Contact**	The arm is moved against gravity and the hand touches the mouth with orwithout finger inserted. Do not mark if the head is on the side and the armis not moved against gravity.<br><br>Score atypical if repetitive.
**Hand-to-Hand Contact**	Both hands are brought together in the midline and the fingers of bothhands repetitively touch, stroke or grasp each other.
	Score atypical if asymmetrical, or if both hands are fisted.
**Fiddling**	The fingers of one or both hands repetitively touch, stroke or grasp someobject, most often the own clothing.<br>Score atypical if the infant touches an object or the own clothing, no finger movements occur and the hand has difficulties to release.
**Reaching**	One or both arms intentionally extend to some object in the immediate environment; the fingers may or may not contact the surface of the object.(This behaviour is not elicited by a tester but occurs spontaneously.)

**Foot-to-Foot Contact**	Legs are elevated and feet are brought together with plantar-to-plantar touching from time to time. Do not mark if the feet remain on the surfaceduring contact. Score atypical if foot-to-foot contact occurs mainly on thetibial side and/or is characterized by repetitive rubbing.
**Legs Lift**	Both legs lift vertically upward; partial or full extension at the knees; hips and pelvis are slightly tilted upward; one or both hands may touch or graspthe knees; sometimes it occurs together with anteflexion of the head.<br><br>Score atypical if it occurs stiff and without variation.
**Segmental Movements of Fingers and Wrists**	Independent movements of fingers and/or movements of moderate speedat the level of the wrist joint including rotation, palmar flexion and extension, and ulnar or radial flexion.<br><br>Score atypical if asymmetrical.
**Arching**	After the soles touch the surface, the infant flexes the back and neckmuscles causing a full trunk and head curve to form. Sometimes locomotion occurs.<br>Do not mark if arching is a sign of discomfort.<br><br>Score atypical if arching is prolonged and/or too stiff.
**Rolling to Side**	As a result of the soles of the feet pushing down on the lying surface, one side of the hip is lifted and rotated. From about 18 weeks onwards, the whole body is turned from supine to prone lying in a movement started bythe head. Sometimes the infant returns to supine lying. Score atypical if the infant moves head and pelvis simultaneously sideways upward, movesthe top leg forward and topples over en bloc, and/or if rolling is non- intentionally.
**Visual Exploration**	The infant visually explores the environment.<br>Score atypical, if abnormal eye movements, (transient) strabismus, nystagmus, and/or setting sun phenomenon occur. Each atypical eye movement pattern is given a separate atypical mark; for instance, if the child has both nystagmus and setting sun phenomenon, give two atypicalscores.
**Hand Regard**	The infant visually attends to the movements of his/her hand(s).
**Head Anteflexion**	The head is moved against gravity, sometimes the chin touches the trunk.Score atypical if prolonged and too stiff.
**Circular Arm Movements**	Uni- or bilateral, monotonous, slow forward rotations of the semi-flexed or extended arms, starting in the shoulder. They occur with our without spread fingers.

**Postural Pattern**	**Definition**
**Head Centered**	The head can be kept centered for at least 10 seconds; chin and sternum are in one line.<br>Score atypical if the head cannot be centered, i.e. is tilted or in lateral position.

**Body Symmetry**	An imaginary line through the shoulder joints and an imaginary line through the hip joints run parallel. Score atypical if this is not the case throughout the recording. 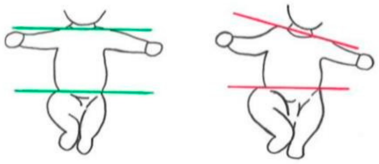
**Asymmetric Tonic Neck (ATN) Posture**	The ATN posture cannot be observed or the extended arm can be easily flexed without turning the head. Score atypical if each spontaneous side movement of the head elicits an ATN that cannot be overcome by flexion of the extended arm.
**Flat Posture**	Lying in supine, all four limbs are mainly on the surface; antigravity movements and flexion in hips and knees are rare; arms and legs hardly move above the level of the trunk.
**Variability of Finger Postures**	Postures of the fingers, which result from isolated movements of one finger, simultaneous movements of two or three fingers, and/or sequential movements of two or more fingers. Fisting might occur from time to time. Score atypical if finger postures are rare and/or lack variability.
**Predominant Fisting**	Score atypical if fisting occurs more than 80% of the observation time.
**Synchronized Opening and Closing of Fingers**	Bilateral simultaneous extension of all fingers away from the palm is followed by bilateral flexion of all fingers towards the palm.
**Finger Spreading**	Unilateral or bilateral abduction and extension of all fingers.
**Hyperextension of Neck and/or Trunk**	Do not mark if the infant focusses his/her attention to an object or a person in the right or left upper corner.
**Extended Arms**	Bilateral predominant extension of the arms on or above the surface.
**Extended Legs**	Bilateral predominant extension of the legs on or above the surface.

Supplementary Table S4. Age-adequate movement repertoire score

	**9 to 11 Weeks PTA**	**12 to 13 Weeks PTA**	**14 to 15 Weeks PTA**	**16 Weeks PTA and older [a]**
**Score 4**	at least four normal movement patterns	at least four normal movement patterns including normal foot-to-foot contact	at least four normal movement patterns including normal foot-to-foot contact **and** normal hand-to hand contact	at least four normal movement patterns including the following three obligatory patterns: normal foot-to-foot contact **and** normal hand-to-hand contact **and** normal legs lift

**Score 2**	three normal movement patterns	at least four normal movement patterns but not foot-to-foot contact	at least four normal movement patterns including normal foot-to-foot contact **or** normal hand-to hand contact	at least four normal movement patterns including only two of the above mentioned obligatory movement patterns
**Score 1**	less than three normal movement patterns	less than four normal movement patterns	normal foot-to-foot contact and normal hand-to-hand contact are not observable	only one of the above mentioned obligatory movement patterns is present or all of them are absent
